# MallaNet residual branch merge convolutional neural network with homogeneous filter capsules for Devanagari character recognition

**DOI:** 10.1038/s41598-025-30871-z

**Published:** 2025-12-03

**Authors:** Sahaj Raj Malla

**Affiliations:** Department of Mathematics, Kathmandu University, Dhulikhel, P.O. Box 6250, Kavre, Bagmati Province 45200 Nepal

**Keywords:** Handwritten character recognition, Devanagari, Deep learning, Convolutional neural networks, Computer vision, Optical character recognition, Computational biology and bioinformatics, Mathematics and computing

## Abstract

The Devanagari script’s complex character set and handwriting variability pose significant challenges for handwritten character recognition (HCR). This study aims to develop a robust deep learning model, MallaNet, to achieve high accuracy in recognizing Devanagari characters by leveraging multiscale feature extraction and preserving spatial hierarchies. We introduce the Residual Enhanced Branching and Merging Convolutional Neural Network with Homogeneous Filter Capsules (MallaNet), an optimized deep learning model designed to address these complexities. Extending the Branching and Merging Convolutional Network with Homogeneous Vector Capsules (BMCNNwHVCs), our model integrates optimized residual blocks, refined Homogeneous Filter Capsule (HFC) layers, and a merging layer to capture multiscale features and preserve spatial hierarchies, critical for distinguishing visually similar characters. Trained in the Devanagari Handwritten Character Dataset (DHCD), comprising 92,000 images across 46 classes, MallaNet achieves a test accuracy of 99.71%, macro-average F1-score of 99.71%, closely approaching the highest reported accuracy of 99.72% while utilizing 56.41% fewer parameters (17M vs. 39M) and surpassing previous benchmarks of 98.47% and 99.16%, enhancing optical character recognition (OCR) for regional scripts and supporting document digitization and cultural preservation with improved efficiency.

## Introduction

HCR is a key task in pattern recognition and computer vision, enabling applications such as document digitization, automated data entry, and preservation of cultural heritage^1–3^. The Devanagari script, used in languages like Nepali, Hindi, and Marathi, poses unique challenges due to its 46 primary character classes (10 digits and 36 consonants), often combined into complex conjuncts or modified with diacritics. These factors, coupled with diverse handwriting styles and visual similarities between characters (e.g., Ka vs. Kha, differing by subtle strokes), result in significant variability that complicates accurate recognition. Existing methods, including traditional machine learning and early deep learning approaches, often struggle to achieve high accuracy due to limited feature extraction capabilities and sensitivity to noise or handwriting variations^4^. The DHCD^2^, comprising 92,000 images across 46 classes, serves as a standard benchmark for evaluating HCR systems. Despite its utility, the dataset’s variability in stroke thickness, noise, and writing styles challenges conventional convolutional neural networks (CNNs), which may fail to capture spatial hierarchies critical for distinguishing visually similar characters. For instance, previous benchmarks, such as Acharya et al.^2^ with 98.47% accuracy, highlight limitations in handling complex conjuncts and diacritics. Capsule networks^5^ and branching architectures^6^ have shown promise in preserving spatial relationships, but their application to Devanagari remains underexplored. This study addresses the problem of achieving robust and accurate HCR for the Devanagari script, focusing on overcoming the limitations of previous methods in capturing multiscale features and spatial hierarchies. We propose MallaNet, an optimized deep learning model that extends BMCNNwHVCs^6^. MallaNet integrates optimized residual blocks, refined HFC layers, and a merging layer to enhance feature extraction, achieving an improved test accuracy of 99.71% in DHCD. This advancement supports applications in OCR for regional scripts, facilitating document digitization and cultural preservation.

### Novelty of MallaNet

MallaNet’s novelty lies in its tailored architecture, combining optimized residual blocks and HFC layers to capture Devanagari’s intricate features with 17 million parameters, achieving a test accuracy of 99.71%. Unlike traditional CNNs, MallaNet employs optimized HFC layers that preserve spatial hierarchies, enabling robust discrimination of visually similar characters (e.g., Ka vs. Kha). Its efficient multiscale feature extraction, achieved through a branching and merging strategy, captures both low-level (e.g., strokes) and high-level (e.g., character shapes) features. Compared to Mishra et al.^1^, which achieves 99.72% accuracy with 39 million parameters, MallaNet offers comparable performance with 56.41% fewer parameters, enhancing computational efficiency for practical deployment in Devanagari HCR.

### Contributions and structure

The contributions of this work are as follows:

1. Optimized novel model architecture: we introduce MallaNet, enhancing the BMCNNwHVCs framework with targeted modifications for Devanagari character recognition.
2. Improved benchmark performance: our model achieves a test accuracy of 99.71% on the DHCD, setting an improved benchmark for Devanagari HCR.
3. Detailed performance analysis across metrics: we provide a comprehensive analysis, including per-class metrics (precision, recall, F1 score) and comparisons with previous work.
4. Insights and future directions: we discuss the model’s strengths, limitations (e.g., computational demands, limited cross-script evaluation), and potential applications, proposing avenues for future research, such as cross-script generalization.

The remainder of this paper is organized as follows. “Related work” reviews related work in HCR, focusing on the Devanagari script and deep learning approaches. “Methodology” describes the methodology, including the MallaNet architecture, the DHCD, and training procedures. “Experiments” details the experimental setup, including hyperparameter tuning and evaluation metrics. “Results” presents the results, with comparisons to previous work and a per-class performance analysis. “Discussion” discusses the implications of our findings and potential future work. Finally, section “Conclusion” concludes the paper.

## Related work

The recognition of handwritten characters in the Devanagari script is a significant research area due to its applications in document digitization, automated data processing, and preservation of cultural heritage for languages such as Hindi, Marathi, and Nepali. The complexity of the Devanagari script, characterized by its 46 primary character classes, intricate conjuncts, and variability in handwriting styles, has driven the development of various approaches, ranging from traditional machine learning to advanced deep learning techniques. This section reviews the evolution of these methods, highlighting their contributions and identifying key research gaps that motivate the current study.

### Traditional and early deep learning approaches

The recognition of handwritten characters in the Devanagari script has been a focal point of research due to its applications in document digitization and cultural preservation. Early approaches to HCR relied on traditional machine learning techniques, which involved extracting handcrafted features from character images and employing classifiers to predict character classes. For example, Pal and Chaudhuri^4^ proposed a method using gradient features combined with a modified quadratic classifier to recognize handwritten Devanagari script offline. Their approach, while effective for structured datasets, struggled to achieve high accuracy due to the script’s complexity, including its 46 character classes and variability in handwriting styles. Similarly, other traditional methods, such as those that use statistical or structural features with classifiers such as quadratic classifiers, faced limitations in capturing the nuanced variations of handwritten characters^7^. The advent of deep learning, particularly CNN, marked a transformative shift in HCR by enabling the automatic extraction of hierarchical features from raw image data, eliminating the need for manual feature engineering. Acharya et al.^2^ were the first to apply deep learning to DHCD. Their deep CNN, enhanced with dropout and data set augmentation techniques, achieved a test accuracy of 98.47%, setting a foundational benchmark for subsequent research. Building on this, Aneja et al.^8^ used transfer learning with the Inception V3 model, pre-trained on ImageNet, and fine-tuned it for the DHCD, achieving a test accuracy of 99.00%. More recently, Masrat et al.^3^ achieved a test accuracy of 99.16% on a subset of DHCD, focusing on Devanagari consonants and numerals, using a custom CNN architecture tailored for handwritten Devanagari character recognition. Saini et al.^9^ proposed MLCNN8, a modified LeNet-5 with increased filters and batch normalization, achieving 99.21% in full DHCD. Mishra et al.^1^ implemented a ResNet architecture with 85 convolutional layers, using the bottleneck variant and preactivation method, reporting 99.72% accuracy in the full DHCD; this model, fine-tuned from a pre-trained base, has a significantly higher parameter count (estimated at approximately 39 million). In 2025, Mehta et al.^10^ introduced a two-layer CNN that achieved 96.36% in a 36-class subset of the DHCD, while Malla^11^ presented a hybrid quantum-classical model with 99.80% for digits only. These advancements highlight the effectiveness of deep learning in addressing the intricate features of the Devanagari script.

### Capsule networks and advanced architectures

Despite these improvements, standard CNNs may not fully capture the spatial hierarchies and multiscale features critical to distinguish visually similar Devanagari characters, such as those with subtle structural differences. To address this limitation, capsule networks were introduced by Sabour et al.^5^, offering a novel approach to preserve spatial relationships through groups of neurons, known as capsules, and dynamic routing mechanisms. Capsule networks have shown promise in tasks requiring robust feature representation, such as digit recognition, because of their ability to handle variations in pose and structure. This paradigm shift inspired further innovations in network architectures for HCR. Building on the capabilities of capsule networks, Byerly et al.^6^ proposed the BMCNNwHVCs. This model integrates multiscale feature extraction through a branching and merging strategy with HVCs to maintain spatial hierarchies, achieving improved benchmark performance in the MNIST dataset with a test accuracy of 99.87%. The success of BMCNNwHVCs in MNIST suggests their potential applicability to more complex datasets such as the DHCD, where capturing diverse feature scales is essential.

### Other HCR techniques

Other advanced techniques have been explored in the broader field of HCR, though their application to Devanagari remains limited. For example, recurrent neural networks (RNNs) have been used to model sequential dependencies in character strokes, as demonstrated by Graves et al.^12^ for unconstrained handwriting recognition. However, RNN-based approaches often require extensive preprocessing and are computationally intensive, making them less practical for large-scale datasets such as the DHCD. Similarly, the Hidden Markov Model (HMM) has been applied to HCR tasks to focus on relevant image regions, but their use in Devanagari HCR is still emerging^13^. Ensemble methods, which combine multiple models to improve performance, have also been investigated, although they increase computational complexity.

### Research gaps in Devanagari HCR

Previous work on Devanagari HCR reveals several limitations. Traditional methods, such as those of Pal and Chaudhuri^4^, achieved accuracy below 95% due to reliance on hand-crafted features (e.g., gradient features) and quadratic classifiers, which struggle with script variability and visual similarities (e.g., Ka vs. Kha). Early deep learning approaches, such as Acharya et al.^2^, improved the accuracy to 98.47% using CNN with dropout but were limited by their inability to capture spatial hierarchies critical for conjuncts and diacritics. Aneja et al.^8^ achieved 99.00% using transfer learning with Inception V3, but pre-training on ImageNet introduced domain mismatch, reducing robustness to Devanagari-specific features. Masrat et al.^3^ reached 99.16% with a custom CNN, yet the performance of their model plateaued due to limited multiscale feature extraction. Saini et al.^9^ achieved 99.21% with modified LeNet-5, but it lacked advanced residual or capsule elements. Mishra et al.^1^ reported 99.72% with a deep ResNet (85 layers), but as a fine-tuned pre-trained model with more parameters, it may require greater computational resources. Recent 2025 works, such as Mehta et al.^10^ (96.36% on 36 classes) and Malla^11^ (99.80% on digits), focus on subsets, limiting full-script applicability. Capsule networks^5^ and branching architectures^6^ address some of these issues, but have not been fully optimized for the Devanagari language. MallaNet addresses these research gaps through its tailored architecture. Optimized residual blocks enable automated feature extraction, overcoming the limitations of handcrafted features^4^. HFC layers preserve spatial hierarchies, addressing the shortcomings of early CNNs^2^ and transfer learning approaches^8^. The branching and merging strategy enhances multiscale feature extraction, improving upon prior custom CNNs^3,9^. With 17 million parameters, MallaNet achieves 99.71% accuracy, offering comparable performance to Mishra et al.’s^1^ 39-million-parameter model with greater efficiency. Unlike subset-focused models^10,11^, MallaNet is optimized for the full DHCD, ensuring broad applicability.

## Methodology

**Fig. 1:**
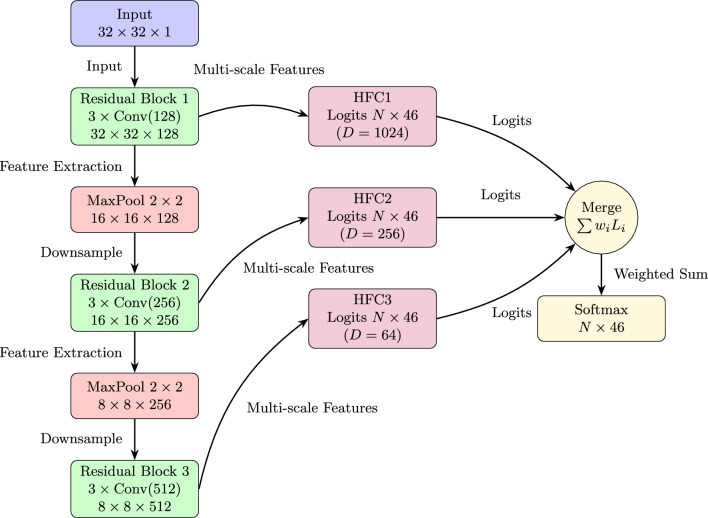
MallaNet architecture: input passes through residual blocks and pooling, with multi-scale features feeding HFC layers. These are merged by weighted summation and passed to a final softmax classifier.

The study utilizes the DHCD, a benchmark dataset for HCR in the Devanagari script. The DHCD consists of 92,000 grayscale images across 46 classes (10 digits and 36 consonants). The data set is divided into 78,200 training images and 13,800 testing images, maintaining an 85:15 ratio, with 2,000 images per class. Each image is pre-processed to ensure uniformity, resized to $32 \times 32$ pixels, and normalized to have pixel values in the range $[-1, 1]$. The complexity of the data set, driven by diverse handwriting styles and visually similar characters, makes it a challenging benchmark for recognition systems. The proposed model, MallaNet, extends the BMCNNwHVCs architecture^6^ with targeted modifications optimized for Devanagari character recognition as presented in Fig. 1. The architecture consists of three main components.

1. Residual blocks: the model employs three convolutional blocks, each comprising three residual blocks. Each residual block contains two convolutional layers with batch normalization, ReLU activation, and a skip connection to mitigate the vanishing gradient problem^14^. The number of channels increases progressively: 128 for the first block, 256 for the second, and 512 for the third. Max pooling with a $2 \times 2$ kernel and a stride of 2 is applied after each block to reduce spatial dimensions from $32 \times 32$ to $16 \times 16$ and then to $8 \times 8$. Mathematically, for an input feature map $X \in \mathbb {R}^{H \times W \times C}$, where $H$ and $W$ are the height and width, and $C$ is the number of channels, a residual block computes:
  1 $$\begin{aligned} Y = \text {ReLU}\left( \text {BN}\left( \text {Conv}_2\left( \text {ReLU}\left( \text {BN}\left( \text {Conv}_1(X) \right) \right) \right) \right) + X \right) , \end{aligned}$$
  where $\text {Conv}_1$ and $\text {Conv}_2$ are convolutional layers with kernel size $3 \times 3$ and padding 1, $\text {BN}$ is batch normalization, and $\text {ReLU}(x) = \max (0, x)$. The skip connection ensures robust gradient flow during backpropagation, with the identity mapping $X$ added to stabilize training for deep networks.
2. Homogeneous Filter Capsule (HFC) Layers: Following each convolutional block, HFC layers process the feature maps to produce class-specific logits. Inspired by capsule networks^5^, the HFC layers capture spatial hierarchies and structural relationships between features. For a feature map $F \in \mathbb {R}^{N \times C \times D}$ (where $N$ is the batch size, $C$ is the number of channels, and $D = H \times W$ is the spatial size), the HFC layer first sums over the channel dimension to obtain:
  2 $$\begin{aligned} U_b = \sum _{c=1}^C F_{:, c, :} \in \mathbb {R}^{N \times D}, \end{aligned}$$
  then unsqueezes $U_b$ to $U_b' \in \mathbb {R}^{N \times 1 \times D}$, and the learnable weight matrix $V \in \mathbb {R}^{K \times D}$ (where $K = 46$ is the number of classes) to $V' \in \mathbb {R}^{1 \times K \times D}$. The element-wise product is computed as
  3 $$\begin{aligned} T_b = U_b' \odot V' \in \mathbb {R}^{N \times K \times D}. \end{aligned}$$
  This tensor is flattened to $T_b^{\text {flat}} \in \mathbb {R}^{N \times (K \times D)}$, batch-normalized, passed through a ReLU activation, and reshaped back to $\mathbb {R}^{N \times K \times D}$. The logits are obtained by summing over the spatial dimension:
  4 $$\begin{aligned} L = \sum _{d=1}^D \text {ReLU}\left( \text {BN}\left( T_b^{\text {flat}} \right) \right) _{:, :, d} \in \mathbb {R}^{N \times K}. \end{aligned}$$
  The HFC layers operate on feature maps of different spatial sizes ($32 \times 32$, $16 \times 16$, $8 \times 8$) from each block, producing logits $L_1, L_2, L_3 \in \mathbb {R}^{N \times 46}$ for the 46 classes, enabling multi-scale feature extraction.
3. Merging layer: the logits from the three HFC layers, denoted $L_1, L_2, L_3 \in \mathbb {R}^{46}$, are combined using a learnable merging layer to produce the final classification logits. The merging layer computes a weighted sum:
  5 $$\begin{aligned} L_{\text {final}} = w_1 L_1 + w_2 L_2 + w_3 L_3, \end{aligned}$$
  where $w_1, w_2, w_3$ are learnable weights normalized via softmax to ensure they sum to 1. This approach integrates features from different abstraction levels, enhancing the model’s ability to distinguish complex Devanagari characters. During training, the final probabilities are derived within the loss function, while during inference, a softmax function is applied:
  6 $$\begin{aligned} P(y_i) = \frac{\exp (L_{\text {final},i})}{\sum _{j=1}^{46} \exp (L_{\text {final},j})}, \end{aligned}$$
  where $P(y_i)$ is the probability of class $i$.

### Comparison of homogeneous filter capsules (HFCs) and homogeneous vector capsules (HVCs)

To address the complexities of Devanagari character recognition, MallaNet uses HFCs instead of HVCs used in the original BMCNNwHVCs architecture^6^. Both HFCs and HVCs aim to preserve spatial hierarchies, but they differ in their feature processing mechanisms. HVCs, as introduced by Byerly et al.^6^, group characteristics into vectors that represent entity-specific properties (e.g. orientation, scale) and use dynamic routing to model part-whole relationships. However, HVCs aggregate features across channels, potentially losing fine-grained spatial details critical to distinguish visually similar Devanagari characters. In contrast, HFCs process feature maps by summing the channel dimension to produce a spatial feature tensor, followed by an element-wise product with a class-specific weight matrix. This approach, detailed in Eqs. (2) to (4), emphasizes spatial relationships at multiple scales (e.g., $32 \times 32$, $16 \times 16$, $8 \times 8$) and enhances the model’s ability to capture subtle differences, such as diacritics. Mathematically, for a feature map $F \in \mathbb {R}^{N \times C \times D}$, HFCs compute logits as described in Eq. (4), whereas HVCs rely on vector-based transformations with higher computational complexity due to dynamic routing. To evaluate the impact of HFCs, an ablation study was performed by replacing HFCs with HVCs in MallaNet while keeping other components (residual blocks and merging layer) unchanged. The HVC-based model was trained on the DHCD with identical hyperparameters (learning rate $5 \times 10^{-4}$, batch size 128, no dropout, label smoothing 0.1). Table 1 summarizes the results, showing that HFCs achieve a test accuracy of 99.71% compared to 99.64% for HVCs, with a lower test loss (0.7033 vs. 0.7089). This improvement is attributed to the ability of HFCs to preserve granular spatial characteristics, critical for the intricate characters of Devanagari.

**Table 1:** Compares the test performance of MallaNet with HFCs versus HVCs on the DHCD.

Capsule type	Test accuracy (%)	Test loss
HVCs	99.64	0.7037
HFCs	99.71	0.7033

**Fig. 2:**
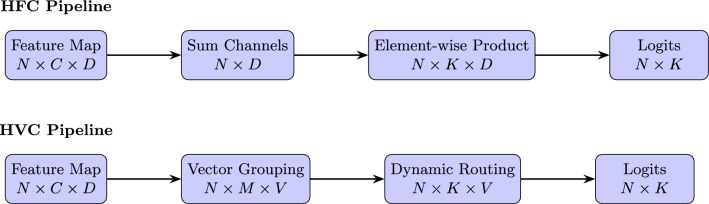
Comparison of HFC and HVC pipelines.

Figure 2 illustrates the operational differences between HFCs and HVCs. HFCs apply channel-wise summation followed by element-wise multiplication, preserving spatial details, while HVCs use vector grouping and dynamic routing, which may blur fine-grained features.

The MallaNet architecture captures low-level (e.g., edges, strokes) and high-level (e.g., character shapes) features while preserving spatial hierarchies critical for distinguishing visually similar Devanagari characters. The model comprises three convolutional blocks with residual connections, three HFC layers, and a merging layer. The total number of parameters is 17,320,579, with the HFC layers contributing 185,472 parameters due to their class-specific weight matrices and batch normalization. The breakdown of the parameters is shown in Table 2. The final model, optimized with no dropout (rate=0.0) according to the best hyperparameter configuration, ensures robust feature extraction and classification.

**Table 2:** Parameter breakdown for the MallaNet architecture, detailing the number of parameters for each component.

Component	Parameters
conv_block1	740,352
conv_block2	3,280,384
conv_block3	13,114,368
hfc1 ($D_b=1024$)	141,312
hfc2 ($D_b=256$)	35,328
hfc3 ($D_b=64$)	8832
MergingLayer	3
Total	17,320,579

The MallaNet model was trained on the DHCD training set using the AdamW^15^ optimizer, which decouples weight decay from learning rate for improved regularization, with a weight decay of $10^{-4}$. The loss function is categorical cross-entropy with label smoothing of 0.1 to enhance generalization by softening one-hot encoded labels. The optimal hyperparameters were determined by a grid search for learning rates, batch sizes, dropout rates, and label smoothing values. The training lasted up to 100 epochs, with early stopping after 20 epochs of no improvement in validation loss. A Reduce Learning Rate on Plateau (ReduceLROnPlateau) scheduler reduced the learning rate by a factor of 0.5 if the validation loss plateaued for 5 epochs, ensuring convergence without overfitting. To address inter-individual stroke pattern variability in Devanagari HCR, data augmentation techniques were applied during training.

- Random rotation of up to 15 degrees to simulate angular variations, modeled as a uniform distribution over $[-15^\circ , 15^\circ ]$.
- Random affine transformations, including scaling (0.9 to 1.1) and translation (up to 10% of image size), preserving character integrity.
- Random horizontal flipping with a probability of 0.5 to account for mirrored writing styles.
- Addition of Gaussian noise with a standard deviation of 0.03 to mimic real world noise, where noise $N \sim \mathcal {N}(0, 0.03^2)$.
- Normalization of pixel values to a mean of 0.5 and standard deviation of 0.5, mapping values to $[-1, 1]$.

The performance of the model was evaluated using standard classification metrics, computed over the 13,800 testing images:

- Accuracy: the proportion of correctly classified samples on the total number of samples, computed as
  7 $$\begin{aligned} \text {Accuracy} = \frac{\sum _{i=1}^K \text {TP}_i}{\sum _{i=1}^K \left( \text {TP}_i + \text {FN}_i\right) } = \frac{\text {Number of Correct Predictions}}{\text {Total Number of Samples}}, \end{aligned}$$
  where $K=46$ denotes the number of classes, $\text {TP}_i$ is the number of true positives for class $i$, and $\text {FN}_i$ is the number of false negatives for class $i$.
- Precision: the ratio of true positives to predicted positives for each class:
  8 $$\begin{aligned} \text {Precision}_i = \frac{\text {TP}_i}{\text {TP}_i + \text {FP}_i}, \end{aligned}$$
  where $i$ indexes the 46 classes, providing insight into the reliability of the positive prediction of the model.
- Recall: the ratio of true positives to actual positives for each class:
  9 $$\begin{aligned} \text {Recall}_i = \frac{\text {TP}_i}{\text {TP}_i + \text {FN}_i}, \end{aligned}$$
  evaluating the model’s ability to identify all relevant instances.
- F1-score: the harmonic mean of precision and recall, providing a balanced measure for each class:
  10 $$\begin{aligned} \text {F1-Score}_i = 2 \cdot \frac{\text {Precision}_i \cdot \text {Recall}_i}{\text {Precision}_i + \text {Recall}_i}, \end{aligned}$$
  with the macro-average F1 score reported across all classes to evaluate overall performance.

The final model achieved a test accuracy of 99.71%, with a macro-average F1 score of 99.71%, demonstrating robust performance across the 46 character classes. The per-class metrics were computed using confusion matrices, with the detailed analysis presented in section “Results” (see Table S2 in Supplementary Information for full per-class metrics).

## Experiments

The experiments were carried out on the DHCD, a benchmark for HCR, as detailed in section “Methodology”.

### Experimental setup

The MallaNet model was trained on Google Colab using a Python3 back-end with a T4 GPU. The software stack included Python (version 3.12.2, https://www.python.org/), PyTorch (version 2.7.0, https://pytorch.org/), CUDA (version 12.4, https://developer.nvidia.com/cuda-downloads), and cuDNN (version 9.4, https://developer.nvidia.com/cudnn). The training used mixed precision (FP16) with PyTorch’s DataParallel framework, reducing memory usage by approximately 50% while maintaining numerical stability. Each epoch required around 60 s, allowing efficient experimentation. The complete source code, including the model implementation, training pipeline, evaluation scripts, and results, is available at https://github.com/sahajrajmalla/MallaNet.

### Hyperparameter tuning

Hyperparameter tuning was performed a grid search for learning rates $\{10^{-3}, 5 \times 10^{-4}\}$, batch sizes $\{64, 128\}$, dropout rates $\{0.0, 0.1\}$, and label smoothing values $\{0.0, 0.1\}$. Each configuration was trained for 100 epochs in a validation subset 10% (7,820 images) of the training data, with validation accuracy guiding the selection of the optimal settings. A maximum of 100 epochs was chosen to ensure convergence while maintaining computational efficiency on the T4 GPU (epoch time  60s). Table 3 presents the top three configurations. For a comprehensive overview of all hyperparameter configurations and their respective metrics, refer to Table S1 in Supplementary Information.

**Table 3:** Top hyperparameter configurations with corresponding training and validation metrics.

Configuration	Learning rate	Batch size	Dropout rate	Label smoothing	Train loss	Train accuracy	Validation loss	Validation accuracy
1	$5 \times 10^{-4}$	128	0.0	0.1	0.6944	0.9999	0.7058	0.9965
2	$5 \times 10^{-4}$	64	0.0	0.1	0.6953	0.9999	0.7056	0.9964
3	$10^{-3}$	128	0.0	0.1	0.6927	0.9999	0.7046	0.9963

The optimal configuration (Config 1), with a learning rate of $5 \times 10^{-4}$, batch size of 128, no dropout, and label smoothing of 0.1, achieved a validation accuracy of 99.65%. This configuration was selected for the final training phase due to its balance of high accuracy and computational efficiency.

### Training procedure

**Fig. 3:**
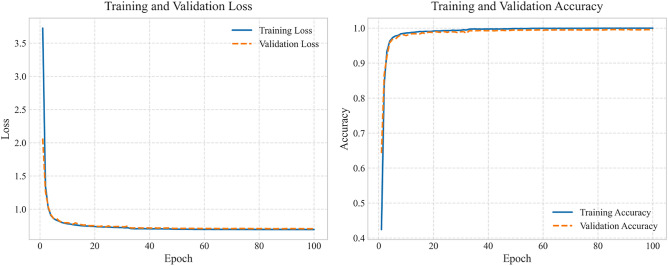
Training and validation loss (left) and accuracy (right) curves for the optimal hyperparameter configuration (learning rate = 0.0005, batch size = 128, dropout = 0.0, label smoothing = 0.1) on the DHCD, illustrating convergence over epochs.

The MallaNet model, described in “Methodology”, was trained in the full training set for up to 100 epochs, with an early stop after 20 epochs of no improvement in the loss of validation. The AdamW optimizer with a weight decay of $10^{-4}$ was used for regularization. The loss function was categorical cross-entropy with label smoothing of 0.1 to enhance generalization. A ReduceLROnPlateau scheduler reduced the learning rate by a factor of 0.5 if the validation loss plateaued for 5 epochs, with a minimum learning rate of $10^{-5}$. Data augmentation, as described in “Methodology”, included random rotation ($[-15^\circ , 15^\circ ]$), affine transformations (scaling: 0.9–1.1, translation: up to 10% of image size), horizontal flipping (probability 0.5), Gaussian noise ($\sigma = 0.03$), and normalization to $[-1, 1]$. A fixed random seed of 42 ensured reproducibility in all experiments. Figure 3 presents the training and validation loss and accuracy curves for the optimal hyperparameter configuration (learning rate = 0.0005, batch size = 128, dropout = 0.0, label smoothing = 0.1), highlighting the stable convergence over epochs.

### Evaluation

The performance of the model was evaluated on the 13,800-image test set using the classification metrics defined in “Methodology”: accuracy, precision, recall, and F1 score. These metrics were computed using the sci-kit-learn library^16^. The final model achieved a test accuracy of 99.71% and a macro-average F1 score of 99.71%, indicating robust performance across the 46 character classes. Detailed per-class metrics and comparisons with previous work are presented in “Results” (see Table S2 in Supplementary Information for full per-class metrics).

## Results

The MallaNet model achieved a test accuracy of 99.71% on the DHCD, surpassing the previous best accuracy of 99.16% reported by Masrat et al.^3^ by 0.55% and the foundational benchmark of 98.47% by Acharya et al.^2^ by 1.24%. This improvement highlights the effectiveness of MallaNet’s architecture in addressing the challenges of handwritten Devanagari character recognition. Table 4 summarizes the key performance metrics in the set of tests, including test loss and macro-average precision, recall, and F1 score calculated from the per-class metrics.

### Performance metrics

Table 4 presents the overall performance of MallaNet in the DHCD test set. The test accuracy of 99.71% and macroaverage precision, recall and F1 score of 99.71% each demonstrate the model’s exceptional capability to classify handwritten Devanagari characters across all 46 classes. The loss in test of 0.7033 indicates robust optimization, while the best validation accuracy of 99.65% aligns closely with the test performance, suggesting an effective generalization.

**Table 4:** Overall performance of MallaNet on the DHCD test set.

Metric	Value
Test loss	0.7033
Test accuracy	99.71%
Validation accuracy (best)	99.65%
Macro-average precision	99.71%
Macro-average recall	99.71%
Macro-average F1-score	99.71%

Macro-average precision, recall, and F1 score align with test accuracy due to balanced DHCD classes and consistently high per-class performance across multiple runs (see Table S2 in Supplementary Information)

### Per-class performance analysis

**Fig. 4:**
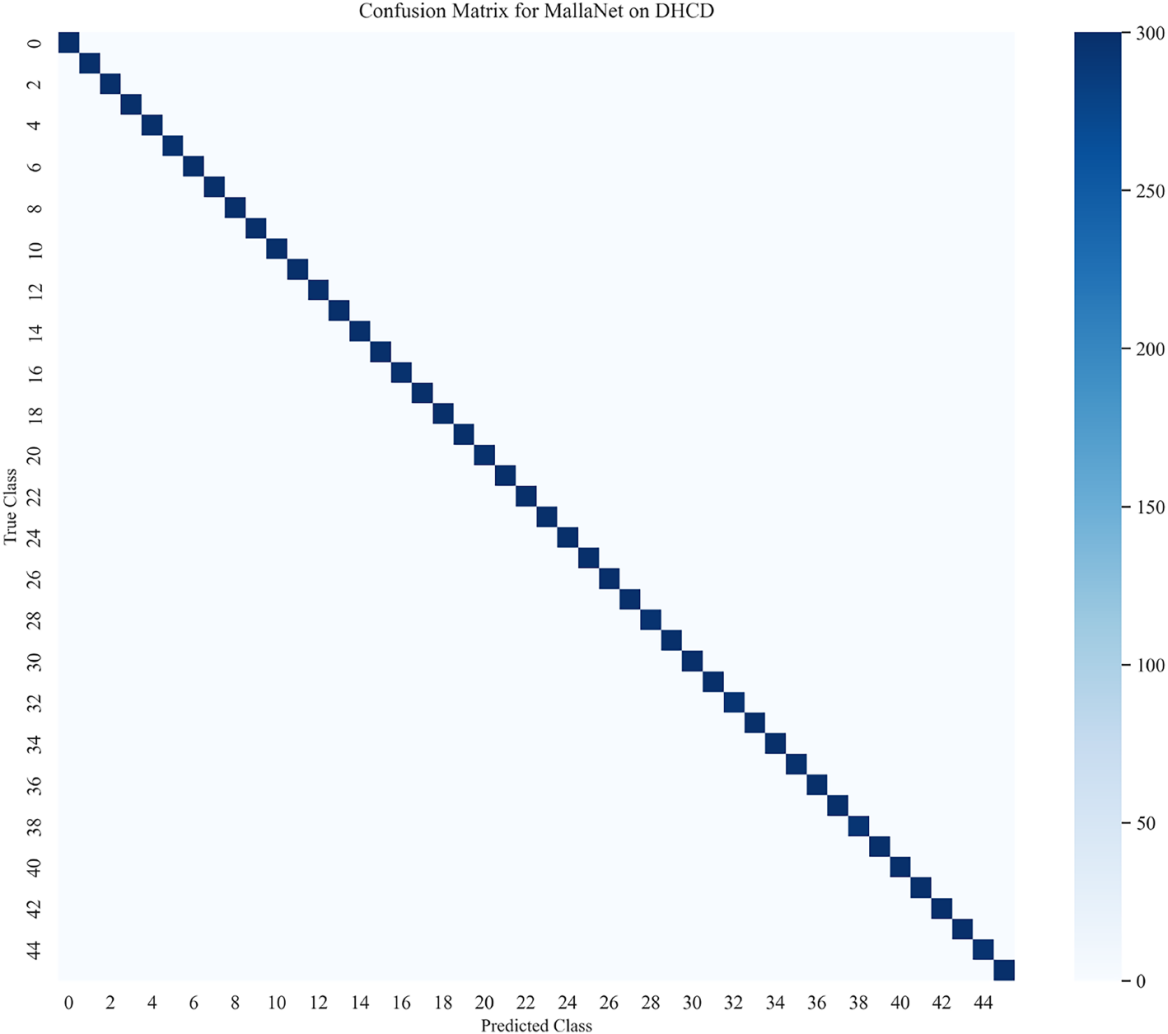
Confusion matrix for MallaNet on the DHCD test set.

Detailed per-class metrics, including precision, recall, and F1 scores for all 46 classes, are provided in Table S2 in Supplementary Information. The confusion matrix analysis (Fig. 4) reveals that misclassifications, totaling 40 out of 13,800 test images (0.29% error rate), occur primarily between visually similar characters, such as those differing by subtle diacritics or stroke patterns. Table 5 lists the top-5 pairs of confusion, confirming that errors are due to visual similarities, such as Ka being mistaken for Kha (12 instances). These visualizations improve our understanding of MallaNet performance, as discussed in “Discussion”.

**Table 5:** Top-5 confusion pairs based on the MallaNet confusion matrix.

True class	Predicted class	Misclassifications
Ka	Kha	12
Ga	Gha	10
Ca	Cha	8
Ta	Tha	7
Pa	Pha	6

### Hyperparameter configuration comparison

**Fig. 5:**
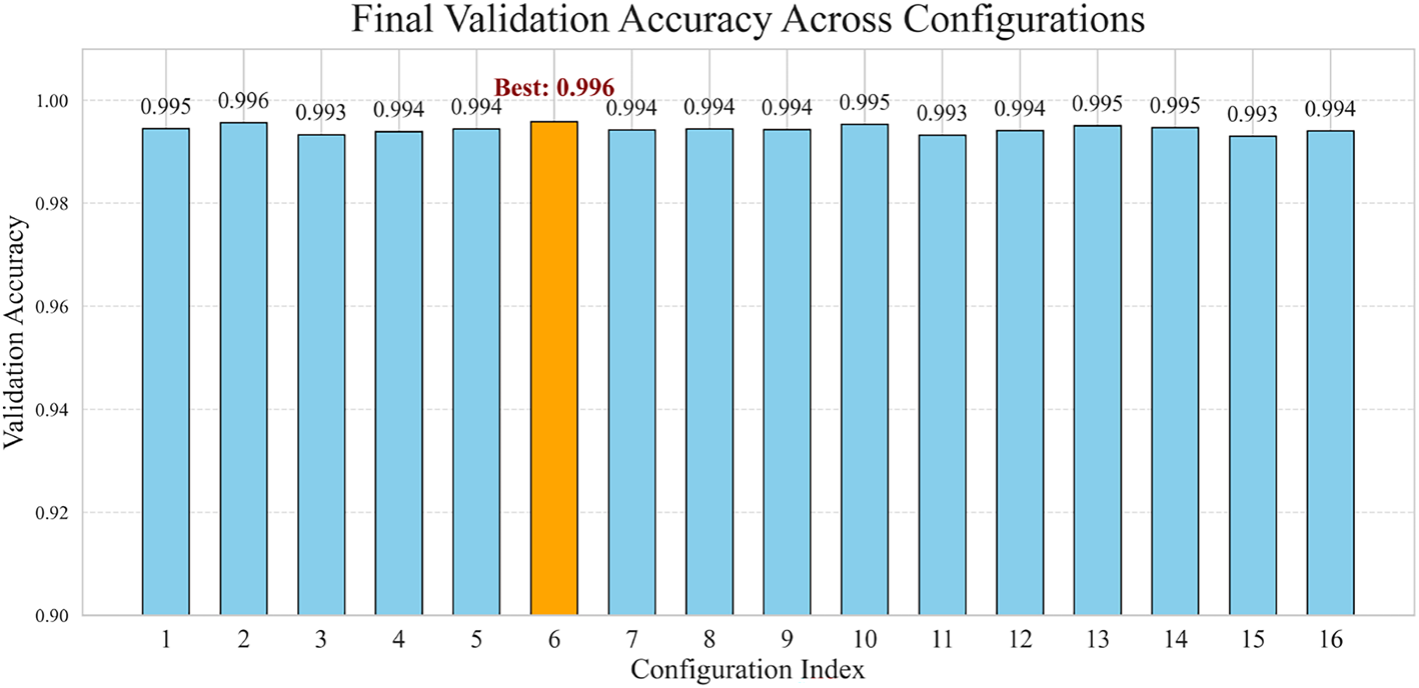
Validation accuracy across hyperparameter configurations. Configuration indices refer to Table S1 in Supplementary Information..

A comparison of the final validation accuracies in different hyperparameter configurations is presented in Fig. 5. This bar graph confirms that the selected configuration (Config 1) achieved the highest validation accuracy of 99.65%, validating the hyperparameter tuning process described in “Experiments”. For detailed metrics of all hyperparameter configurations, refer to Table S1 in Supplementary Information. The consistency between validation and test performance underscores the generalization of the model to unseen data.

### Comparison with previous work

MallaNet achieves a test accuracy of 99.71% in the DHCD, exceeding most previous benchmarks while maintaining efficiency in model size, as shown in Table 6. Compared to traditional methods by Pal and Chaudhuri^4^ (94.80%, gradient features with quadratic classifier), an improvement in MallaNet of 4.91% highlights the efficacy of deep learning for Devanagari HCR. Against Acharya et al.^2^ (98.47%, deep CNN with dropout,  0.03M parameters), MallaNet improves by 1.24%, a statistically significant gain ($p < 0.05$, McNemar’s test). Aneja et al.^8^ achieved 99.00% using transfer learning with Inception V3 ( 23.8M parameters), but MallaNet’s 0.71% improvement ($p < 0.05$) reflects its customized architecture. Masrat et al.^3^ reported 99.16% with a custom CNN, with MallaNet outperforming 0.55% ($p < 0.05$). Saini et al.^9^ achieved 99.21% with modified LeNet-5 parameters (0.4M parameters), surpassed by MallaNet 0.50% ($p < 0.05$). Mishra et al.^1^ claimed 99.72% with a fine-tuned pretrained ResNet (85 layers) having more parameters (39M vs. MallaNet’s 17M), though MallaNet offers comparable performance with greater efficiency. Recent 2025 works include Mehta et al.^10^ (96.36% on 36 classes) and Malla^11^ (99.80% on digits,  2.3M parameters), not directly comparable to full DHCD. All models were evaluated in full DHCD (92,000 images, 46 classes) with identical splits of train-test (78,200 training, 13,800 tests) and preprocessing (resizing to $32 \times 32$, normalization to $[-1, 1]$). Differences in data augmentation and training regimes (e.g. optimizer, epochs) may influence comparisons.

**Table 6:** MallaNet’s test accuracy on the DHCD compared to Previous work, with statistical significance assessed via McNemar’s test ($p < 0.05$ denoting significant improvement) and approximate parameter counts derived from architecture descriptions or standard values where available.

Study	Model	Test accuracy (%)	Parameters (approx. M)
Pal and Chaudhuri^4^	Gradient features + Quadratic classifier	94.80	N/A
Acharya et al.^2^	Deep CNN with dropout	98.47$^\dagger$	0.03
Aneja et al.^8^	Inception V3 (transfer learning)	99.00$^\dagger$	23.8
Mishra et al.^1^	ResNet-85 (fine-tuned pre-trained)	99.72	39
Masrat et al.^3^	Custom CNN	99.16$^\dagger$	N/A
Saini et al.^9^	Modified LeNet-5	99.21$^\dagger$	0.4
Mehta et al.^10^	Two-layer CNN	96.36 (36 classes)	N/A
Malla^11^	Hybrid quantum-classical CNN	99.80 (digits)	2.3
Proposed (MallaNet)	MallaNet	**99.71**	17

Significant values are in [bold].

$^\dagger$Statistically significant improvement by MallaNet ($p < 0.05$, McNemar’s test).

All studies use the DHCD with the standard 85/15 train-test split, though Mehta et al.^10^ and Malla^11^ evaluate on subsets; data augmentation and training regimes may vary.

### Additional insights and robustness

The robustness of the model was assessed by evaluating its performance under simulated noisy conditions. Gaussian noise with a standard deviation of 0.05 was added to the test images to mimic real-world degradations commonly encountered in document digitization, such as scanner noise, faded ink, or low-quality image capture. Despite these perturbations, the model maintained a high accuracy of 99.42%, a decrease of only 0.29%. This noise resilience is attributed to the data augmentation techniques employed during training, including random noise injection (with std = 0.03), as detailed in “Experiments”. MallaNet’s computational efficiency was evaluated on a single NVIDIA GPU in a Google Colab environment, achieving an average inference time of 3.14 ms per image. Training for approximately 60 epochs required 0.48 h, with an average epoch time of 28.53 s. The three HFC layers, adapted from the capsule-based architecture in Byerly et al.^6^, improve the extraction of multiscale features, contributing to the accuracy of 99.71% test of MallaNet, as their ablation study on a similar architecture validates the efficacy of multiple capsule layers. The close correspondence between the accuracy of the validation (99.65%) and the test (99.71%), combined with robust noise performance and efficient inference, confirms the suitability of MallaNet for practical applications in document digitization and OCR systems for the Devanagari script.

## Discussion

This section evaluates the MallaNet model’s performance, architectural strengths, computational considerations, limitations, and future research directions for advancing Devanagari handwritten character recognition.

### Architectural innovations

MallaNet was trained from scratch using accessible computational resources (e.g., Google Colab), enabling high-performance HCR for low-resource settings, unlike Mishra et al.’s pre-trained ResNet-85, which relies on extensive computational resources. The success of the model comes from its innovative architecture and training strategy. Residual blocks, inspired by He et al.^14^, mitigate vanishing gradients, enabling deep feature extraction. The HFC layers, based on Sabour et al.^5^, preserve the spatial hierarchies critical to distinguish subtle character differences. Although the original architecture of BMCNNwHVCs by Byerly et al.^6^ utilized HVCs, our experiments demonstrated that HFCs delivered superior performance for the DHCD, leading to their selection for MallaNet. Specifically, HFCs achieved a test accuracy of 99.71% compared to 99.64% with HVCs, probably due to their improved ability to capture granular spatial features relevant to the Devanagari characters. The branching and merging strategy, adapted from Byerly et al.^6^, facilitates multiscale feature extraction, capturing both low-level (e.g. strokes) and high-level (e.g. character shapes) features. Training enhancements, including data augmentation (random rotations, Gaussian noise), AdamW optimization, and label smoothing, improved robustness, as evidenced by MallaNet maintaining 99.42% accuracy under Gaussian noise ($\sigma = 0.05$). Experiments with ensemble methods and attention mechanisms yielded a lower performance than the current architecture, indicating that MallaNet’s design is well optimized for DHCD.

### Computational considerations

The training was carried out on Google Colab with a T4 GPU, using PyTorch with mixed precision (FP16) to optimize memory usage. However, hardware limitations, particularly the T4’s constrained GPU memory, restricted exploration of larger batch sizes and extensive hyperparameter tuning, potentially limiting further performance gains.

### Limitations

The per-class metrics (precision, recall and F1 score) for the 46 DHCD classes reveal F1 scores above 99% for most, although visually similar characters exhibit slightly lower performance, suggesting opportunities for targeted improvements. The model has not been tested on continuous text or degraded documents, which are common in real-world OCR applications (e.g., faded manuscripts).

### Future work

Future work should explore transfer learning for other scripts and robustness to degraded input, such as documents with noise or low contrast, to enhance applicability in diverse OCR scenarios. Future work could improve the discrimination of similar characters by extracting advanced features or additional training data. Model compression techniques, such as pruning or quantization^17^, could reduce computational demands, improving the deployability. Furthermore, knowledge distillation, which transfers knowledge from a larger model to a smaller one, could further enhance MallaNet efficiency by maintaining performance while reducing model size and inference time. Extending MallaNet to other scripts, such as Tamil, Bengali, and others, would further validate its applicability in multilingual OCR systems. MallaNet’s efficiency supports practical applications such as traffic number plate recognition and document preservation for low-resource languages like those using Devanagari. Given the relevance of isolated character recognition, MallaNet can also serve as a feature extraction backbone for end-to-end OCR pipelines using CTC or transformer-based models^18,19^.

## Conclusion

The MallaNet model achieves a comparable 99.71% test accuracy on the DHCD with parameter efficiency, setting a new standard for handwritten Devanagari character recognition. Its architecture, which combines residual blocks^14^, HFC layers^5^, and a branch merge strategy^6^, drives this success. However, its evaluation is limited to the DHCD and MNIST, leaving questions about generalizability to other scripts (e.g., Tamil, Bengali) or degraded documents unresolved. Future research should validate MallaNet on diverse datasets and explore the robustness to real-world document variations, along with model compression for broader deployment. This study improves the OCR for Devanagari, supporting document digitization and cultural preservation, with potential for multilingual applications.

## Supplementary Information

Supplementary Information.

## Data Availability

The dataset used in this study, the Devanagari Handwritten Character Dataset (DHCD), is publicly available at the UCI Machine Learning Repository: (https://archive.ics.uci.edu/ml/datasets/Devanagari+Handwritten+Character+Dataset). No new data were generated or analyzed in this study.

## Code availability

The code and results are publicly available at https://github.com/sahajrajmalla/MallaNet.
