# Supplementary material for: MallaNet residual branch merge convolutional neural network with homogeneous filter capsules for Devanagari character recognition
Source: Sci Rep. 2025 Dec 3;16:1175. doi: 10.1038/s41598-025-30871-z (PMC12789527; doi:10.1038/s41598-025-30871-z)
Supplement: Supplementary file 1 — Supplementary Information. [file 41598_2025_30871_MOESM1_ESM.pdf]

Supplementary Information for MallaNet Residual Branch Merge Convolutional Neural Network with Homogeneous Filter Capsules for Devanagari Character Recognition

1 Supplementary Appendix

1.1 Hyperparameter Tuning Results

Table S1 presents the full hyperparameter tuning results for all 16 configurations tested using Optuna. The best-performing configuration (highest validation accuracy = 0.99648) is highlighted in bold.

Table 1 Full hyperparameter tuning results with training and validation metrics.

Index	LR	Batch Size	Dropout	Label Smoothing	Train Loss	Train Acc	Val Loss	Val Acc
1	0.0010	64	0.0	0.0	0.0006	0.99994	0.0218	0.99601
2	0.0010	64	0.0	0.1	0.6946	0.99992	0.7065	0.99592
3	0.0010	64	0.1	0.0	0.0015	0.99954	0.0241	0.99471
4	0.0010	64	0.1	0.1	0.6966	0.99983	0.7095	0.99523
5	0.0010	128	0.0	0.0	0.0007	0.99991	0.0211	0.99562
6	0.0010	128	0.0	0.1	0.6937	0.99993	0.7046	0.99632
7	0.0010	128	0.1	0.0	0.0014	0.99974	0.0251	0.99434
8	0.0010	128	0.1	0.1	0.6968	0.99988	0.7095	0.99512
9	0.0005	64	0.0	0.0	0.0007	0.99993	0.0201	0.99562
10	0.0005	64	0.0	0.1	0.6953	0.99992	0.7056	0.99642
11	0.0005	64	0.1	0.0	0.0012	0.99984	0.0245	0.99483
12	0.0005	64	0.1	0.1	0.6977	0.99982	0.7094	0.99523
13	0.0005	128	0.0	0.0	0.0006	0.99994	0.0198	0.99583
**14**	**0.0005**	**128**	**0.0**	**0.1**	**0.6944**	**0.99992**	**0.7058**	**0.99648**
15	0.0005	128	0.1	0.0	0.0013	0.99974	0.0254	0.99423
16	0.0005	128	0.1	0.1	0.6973	0.99983	0.7105	0.99534

1.2 Detailed Test Metrics for the Best Model

The best-performing model (Index 14) has the following hyperparameters: learning rate = 0.0005, batch size = 128, dropout = 0.0, and label smoothing = 0.1. This model achieved a test accuracy of 99.71% (test loss = 0.7033) on the official DHCD test set.

Table S2 reports per-class precision, recall, and F1-score for all 46 classes.

Table 2 Per-class precision, recall, and F1-score of the best MallaNet model on the DHCD test set.

Class	Precision	Recall	F1-Score
0	1.0000	1.0000	1.0000
1	1.0000	0.9967	0.9983
2	0.9967	1.0000	0.9983
3	1.0000	0.9967	0.9983
4	1.0000	1.0000	1.0000
5	1.0000	0.9933	0.9967
6	0.9967	0.9967	0.9967
7	1.0000	1.0000	1.0000
8	0.9967	1.0000	0.9983
9	1.0000	1.0000	1.0000
Ka	1.0000	1.0000	1.0000
Kha	1.0000	1.0000	1.0000
Ga	1.0000	1.0000	1.0000
Gha	0.9901	1.0000	0.9950
Nga	1.0000	1.0000	1.0000
Ca	0.9934	1.0000	0.9967
Cha	1.0000	0.9933	0.9967
Ja	1.0000	1.0000	1.0000
Jha	0.9967	1.0000	0.9983
Nya	1.0000	1.0000	1.0000
Ta	1.0000	0.9900	0.9950
Tha	1.0000	1.0000	1.0000
Da	0.9967	0.9967	0.9967
Dha	0.9967	0.9967	0.9967
Na	1.0000	1.0000	1.0000
Ta	0.9771	0.9967	0.9868
Tha	0.9934	1.0000	0.9967
Da	0.9934	0.9967	0.9950
Dha	0.9966	0.9867	0.9916
Na	1.0000	0.9967	0.9983
Pa	1.0000	0.9967	0.9983
Pha	1.0000	1.0000	1.0000
Ba	0.9866	0.9833	0.9850
Bha	1.0000	0.9967	0.9983
Ma	0.9967	1.0000	0.9983
Ya	1.0000	0.9933	0.9967
Ra	0.9967	0.9967	0.9967
La	1.0000	1.0000	1.0000
Va	0.9801	0.9833	0.9817
Sha	1.0000	0.9967	0.9983
Ssa	1.0000	1.0000	1.0000
Sa	0.9934	1.0000	0.9967
Ha	0.9934	1.0000	0.9967
Ksha	1.0000	0.9967	0.9983
Tra	0.9966	0.9867	0.9916
Jna	1.0000	1.0000	1.0000
